# Supplementary material for: Modelling Skylarks (Alauda arvensis) to Predict Impacts of Changes in Land Management and Policy: Development and Testing of an Agent-Based Model
Source: PLoS One. 2013 Jun 6;8(6):e65803. doi: 10.1371/journal.pone.0065803 (PMC3675089; doi:10.1371/journal.pone.0065803)
Supplement: Supporting Information S4 — The skylark ODdox as a zipped archive. (ZIP) [file pone.0065803.s004.zip › Skylark_ODdox/globals_0x6d.html]

ALMaSS Skylark ODdox: File Members


ALMaSS Skylark ODdox
 2.0


- Main Page
- Related Pages
- Classes
- Files

- File List
- File Members

- All
- Functions
- Variables
- Typedefs
- Enumerations
- Enumerator
- Macros

- \_
- a
- b
- c
- d
- e
- f
- g
- h
- i
- j
- k
- l
- m
- n
- o
- p
- r
- s
- t
- u
- v
- w
- x

Here is a list of all file members with links to the files they belong to:

### - m -

- m\_polymapping
  : Landscape.cpp
  , landscape.h
  , rastermap.h
- main()
  : ALMaSS\_CmdLine.cpp
- MapDumpColours
  : misc.cpp
  , skylarks\_all.cpp
- MapErrorState
  : maperrormsg.h
- March
  : landscape.h
- marchfirst
  : plants.h
- MaxFeedRain
  : skylarks\_all.cpp
- MaxNoInflections
  : plants.h
- May
  : landscape.h
- MD\_Hatch
  : skylarks\_all.cpp
- MD\_Threshold
  : skylarks\_all.cpp
- MeanExtractionRatePerMinute
  : skylarks\_all.cpp
- MeanHatchingWeight
  : skylarks\_all.cpp
- molluscicide
  : treatment.h
- mow
  : treatment.h


- Generated on Thu Jan 10 2013 13:15:39 for ALMaSS Skylark ODdox by
   1.8.1.1
